# Supplementary figures and images for: CDKN2A Gene Mutations: Implications for Hereditary Cancer Syndromes
Source: Biomedicines. 2023 Dec 18;11(12):3343. doi: 10.3390/biomedicines11123343 (PMC10741544; doi:10.3390/biomedicines11123343)

## Patient 1

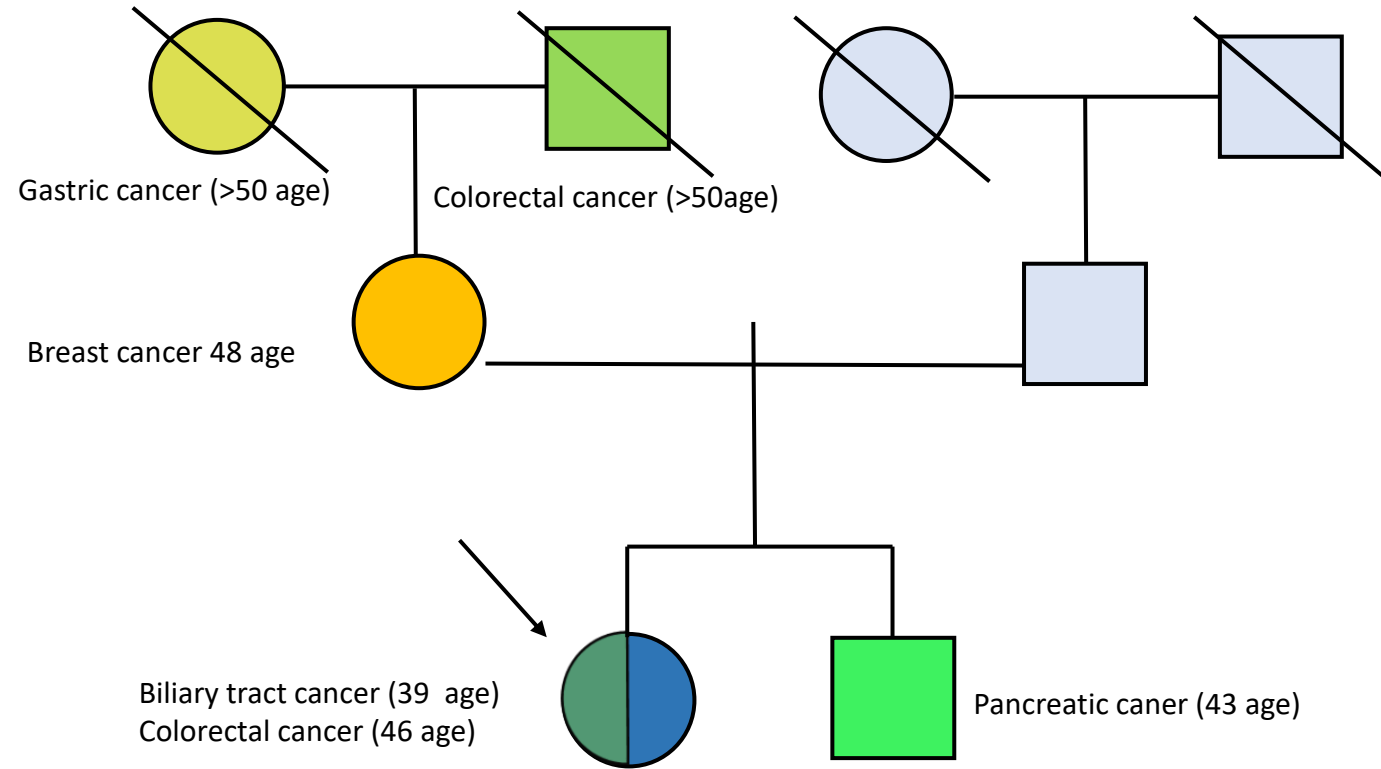

Patient 2

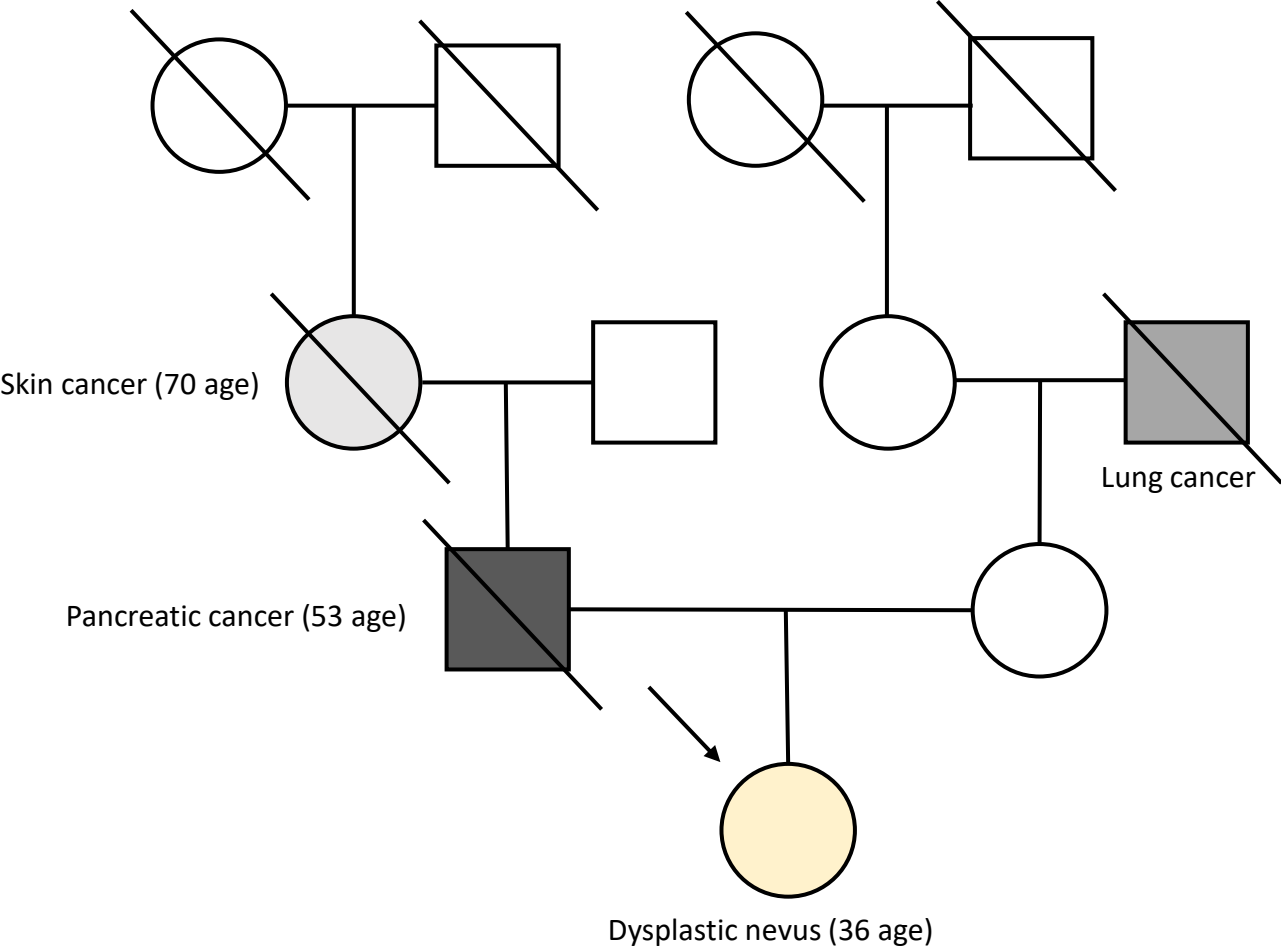

Patient 3

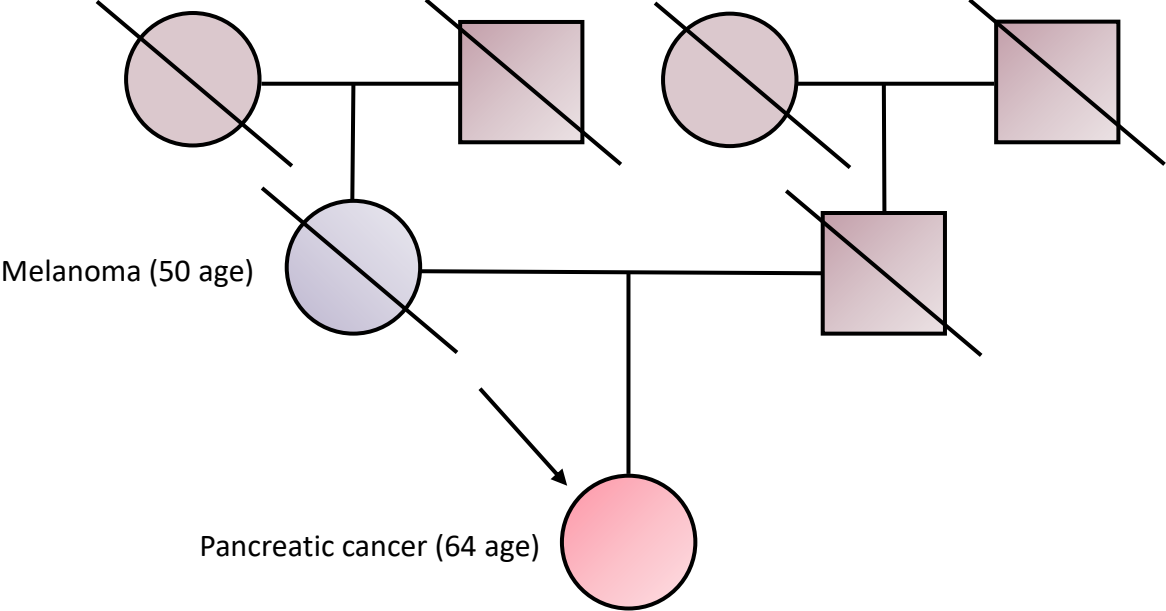

Patient 4

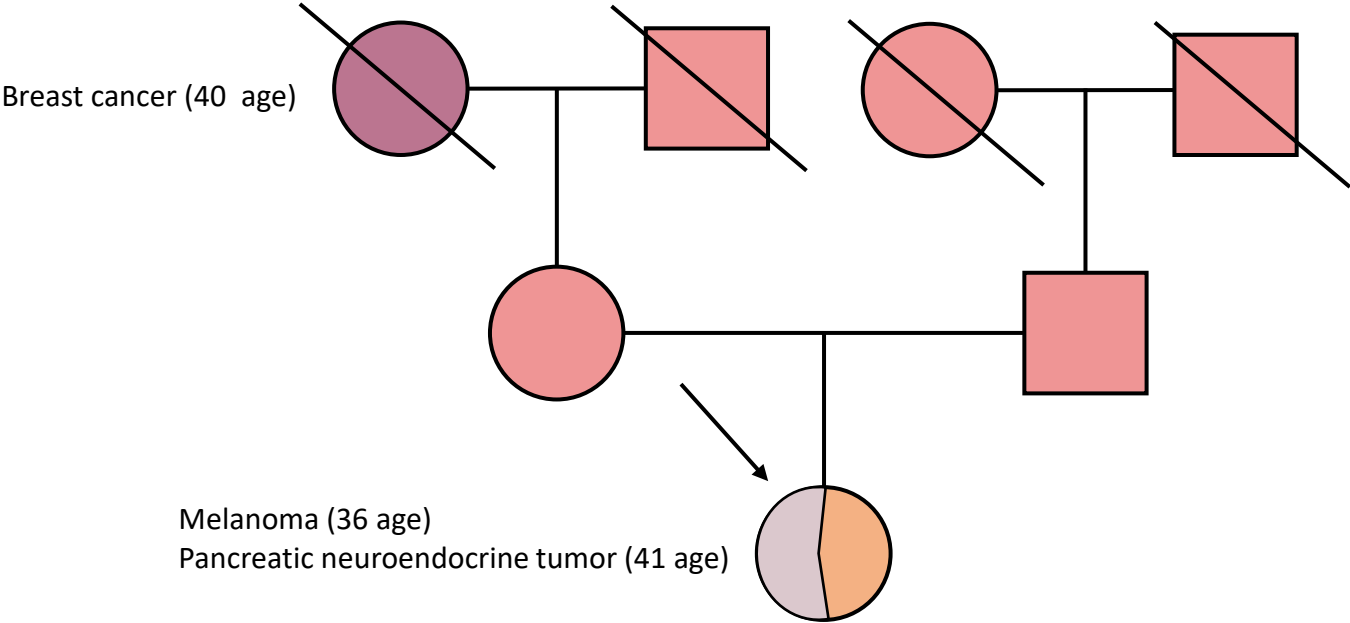

Patient 5

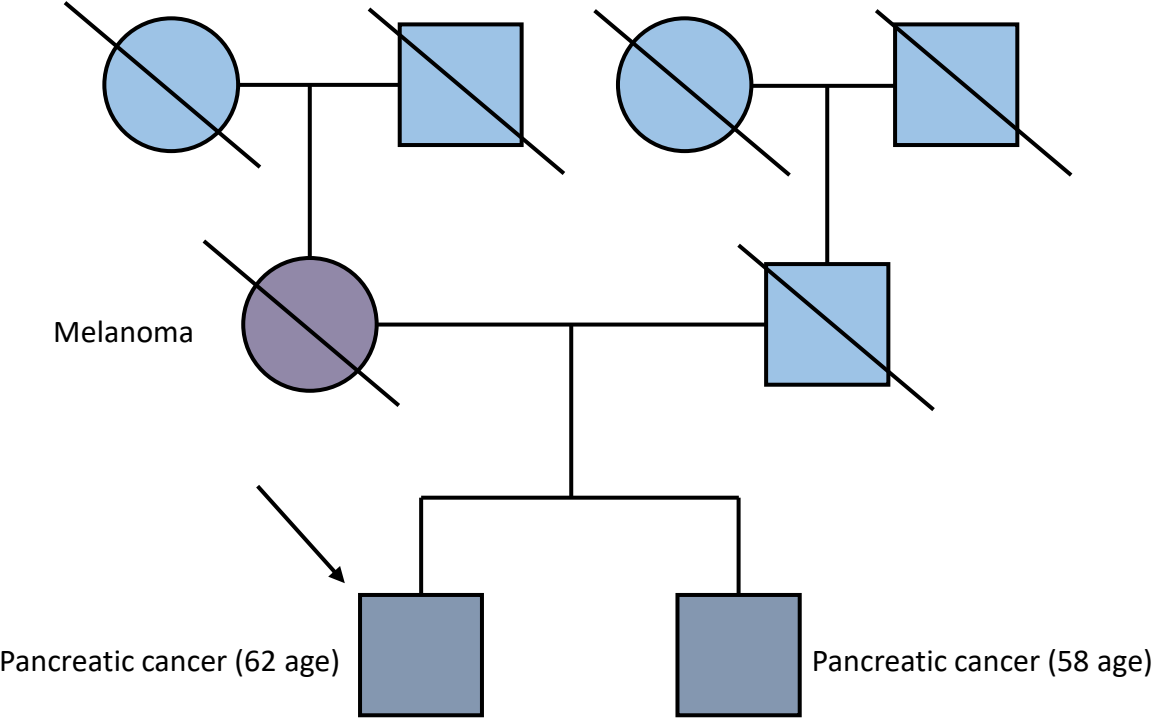

Patient 6

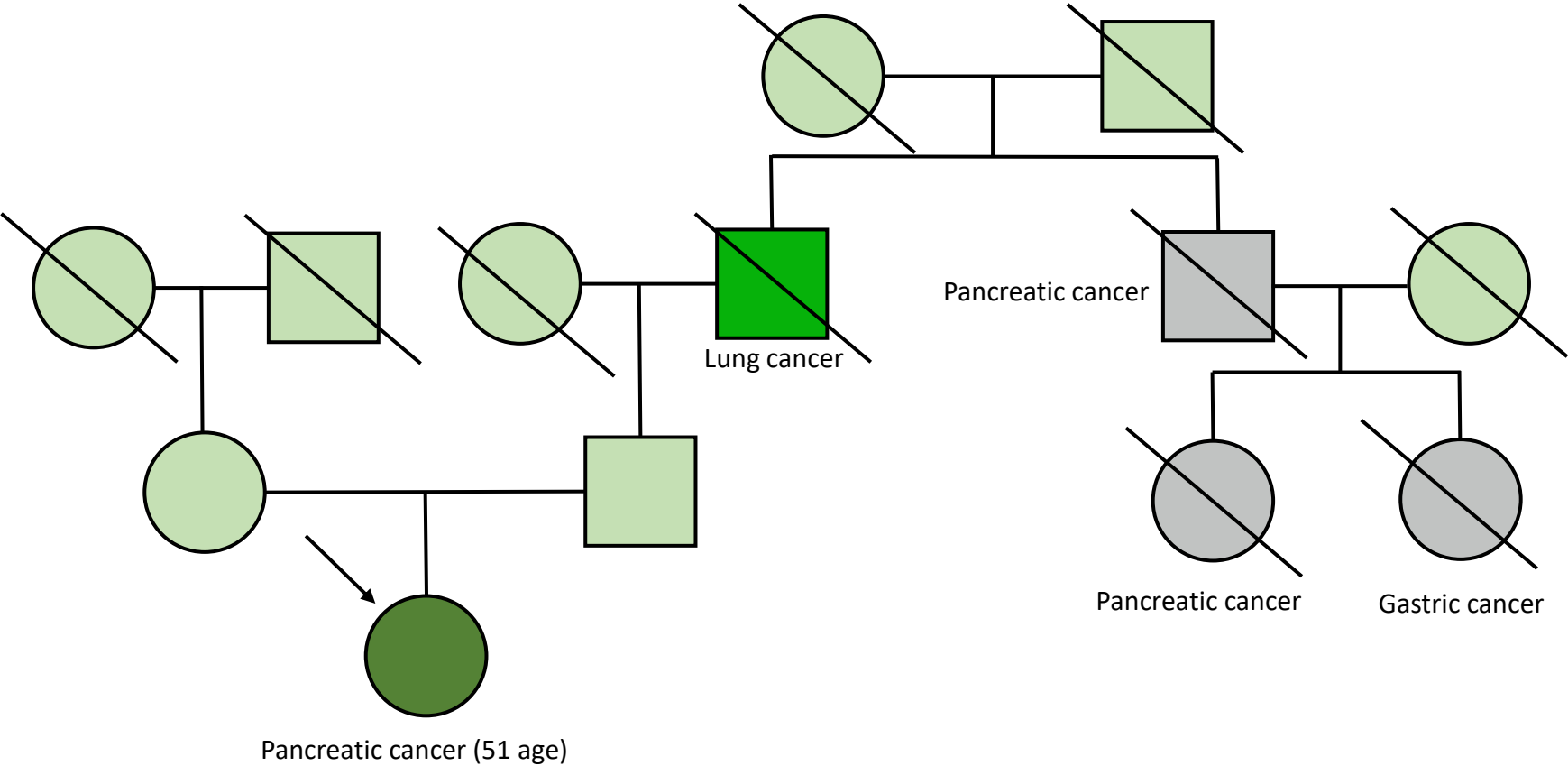

Supplement: Supplementary file 1 [file biomedicines-11-03343-s001.zip › biomedicines-2742408-supplementary.pdf]
